# Towards the Improvement of Ornamental Attributes in Chrysanthemum: Recent Progress in Biotechnological Advances

**DOI:** 10.3390/ijms232012284

**Published:** 2022-10-14

**Authors:** Manjulatha Mekapogu, Oh-Keun Kwon, Hyun-Young Song, Jae-A Jung

**Affiliations:** Floriculture Research Division, National Institute of Horticultural and Herbal Science, Rural Development Administration (RDA), Wanju-gun 55365, Jeollabuk-do, Korea

**Keywords:** breeding, chrysanthemum, floral attributes, genetic engineering, ornamental plants, resistance mechanisms

## Abstract

Incessant development and introduction of novel cultivars with improved floral attributes are vital in the dynamic ornamental industry. Chrysanthemum (*Chrysanthemum morifolium*) is a highly favored ornamental plant, ranking second globally in the cut flower trade, after rose. Development of new chrysanthemum cultivars with improved and innovative modifications in ornamental attributes, including floral color, shape, plant architecture, flowering time, enhanced shelf life, and biotic and abiotic stress tolerance, is a major goal in chrysanthemum breeding. Despite being an economically important ornamental plant, the application of conventional and molecular breeding approaches to various key traits of chrysanthemum is hindered owing to its genomic complexity, heterozygosity, and limited gene pool availability. Although classical breeding of chrysanthemum has resulted in the development of several hundreds of cultivars with various morphological variations, the genetic and transcriptional control of various important ornamental traits remains unclear. The coveted blue colored flowers of chrysanthemums cannot be achieved through conventional breeding and mutation breeding due to technical limitations. However, blue-hued flower has been developed by genetic engineering, and transgenic molecular breeding has been successfully employed, leading to substantial progress in improving various traits. The recent availability of whole-genome sequences of chrysanthemum offers a platform to extensively employ MAS to identify a large number of markers for QTL mapping, and GWAS to dissect the genetic control of complex traits. The combination of NGS, multi-omic platforms, and genome editing technologies has provided a tremendous scope to decipher the molecular and regulatory mechanisms. However, the application and integration of these technologies remain inadequate for chrysanthemum. This review, therefore, details the significance of floral attributes, describes the efforts of recent advancements, and highlights the possibilities for future application towards the improvement of crucial ornamental traits in the globally popular chrysanthemum plant.

## 1. Introduction

The ornamental plant industry has transformed drastically due to increasing global trade. Ornamental plants are extensively used for cut flowers, landscaping, potted flowers, and home gardening [1]. The international ornamental plant industry has increased in volume, value of production, specification, and commercialization. The global production value of ornamental plants and flowers is estimated at USD 36.5 million in 2021. Floriculture significantly influences the horticulture industry, and the global cut flower market is valued at USD 17.6 million, constituting about one-third of the global market value of ornamental plants [2]. The major ornamental flowers that dominate the global cut flower market include rose, chrysanthemum, carnation, orchids, gerbera, freesia, lilies, gladiolus, and ranunculus.

*Chrysanthemum morifolium* is a perennial herbaceous plant belonging to the Asteraceae family, the second most popular cut flower after rose, and a commercially important ornamental plant across the global flower market [3]. Flowers of some species, including *Chrysanthemum morifolium* and *Chrysanthemum indicum*, are rich in nutritive and biologically active components and have been used in medicinal tea and for cosmetic purposes, respectively [4]. It has been cultivated since the 15th century BC as a herb in China [5]. The ancestry of chrysanthemum is unknown, but modern chrysanthemum plants have evolved as a result of intercrossing between naturally occurring species in China and Japan, including *C. japonicus*, *C. indicum*, *C. makinoi*, *C. erubescens*, *C. ornatum*, and *C. sinense* [6]. This genus comprises 100–200 species with varying morphological attributes and has a complex evolutionary history, exhibiting significant variations in morphology and ploidy (2n = 2x = 18, 2n = 36, 54, 72, and up to 90). Cultivated chrysanthemum is generally a complex hexaploid that also shows aneuploidy, with the most frequent and stable conformation of 2n = 6x = 54 [7]. It is known for its beautiful flowers, vibrant color, and diverse floral types and shapes. The high ornamental value of chrysanthemum is due to its rich diversity in flower shape and color, and the highly diverse flower architecture is due to its allohexaploid background and huge genomic complexity [8]. Chrysanthemum has been selected, bred, and developed in various countries, resulting in the commercialization of thousands of cultivars globally with numerous flower colors and shapes, making it the world’s most popular ornamental plant [9].

Flowers are essential and dynamic organs that impart striking attributes to plants through their beautiful colors and morphology. In chrysanthemum, the flower head or capitulum, constituting two types of morphologically different florets, is the main ornamental attribute. The central disc florets in yellow or green colors are hermaphroditic, and the ray florets are male-sterile, exhibiting a diverse range of colors and patterns specific to a cultivar. Chrysanthemum flowers have various shapes, including single, double, pompon, anemone, windmill, incurve, spoon, and pine needles, based on various combinations of floret number, petal size, and floral organ fusion, which are cultivar-dependent [10]. Based on the type of cultivation and growth habits, chrysanthemums are grown as cut-flower types, including spray and standard disbud-cut, pot-flower, ground cover, and traditional [11]. Globally, chrysanthemum is known for its wide cultivation and production of cut flowers and is used as a potted plant for gardening, and ornamental landscaping [12]. Since chrysanthemums represent the top ornamental crop worldwide, introducing novelties with varied ornamental attributes into the market is vital to sustain global competitiveness in the most dynamic sector of floriculture. In addition to the floral color, the ornamental traits of chrysanthemums include flower type, shape, floral scent, flowering time, vase life, and biotic and abiotic stress resistance. The increasing need for floriculture requires the development of novel plant cultivars with elite traits of all ornamental attributes [13,14]. Apart from conventional breeding, recent advances in phenotyping, genotyping, marker development, and multi-omic technologies have been crucial in the breeding and development of novel chrysanthemum cultivars. As a potential substitute, genetic engineering provides a promising platform for developing cultivars with improved ornamental attributes and plant quality. Furthermore, genome editing technologies and their applications, with precise manipulation of genomic sequences at the gene of interest, have provided a powerful platform with greater possibilities of improving desired traits in chrysanthemum. This review outlines the efforts of recent advancements to develop and enhance valuable morphological characteristics resulting in novel varieties of this globally dominant ornamental crop.

## 2. Importance of Floral Attributes

Floral attributes, including the attractive colors and shapes of flowers, leaves, and fruits, floral scent, leaf texture, variegation, and plant architecture, impart beauty and aesthetic value to ornamental plants. All these floral characteristics exert individual or synergetic influences. In addition to aesthetic gratification, floral traits are vital for plant survival. Along with their ornamental value, ornamental plants, including chrysanthemum, contain nutritional and bioactive compounds that are used in medicine and cosmetic industries [4]. The visual quality of ornamental plants, especially cut flowers and potted plants, is crucial for customer choice and commercial value. Hence, ensuring quality traits is crucial to increasing market demand [15]. In addition, customer preference and market demand for ornamental traits in chrysanthemum continue to change rapidly, which compels breeders to develop novel cultivars with improved attributes [10].

In chrysanthemum, cultivar propagation is primarily achieved by vegetative cuttings and suckers. Considerable inbuilt genetic variation confers the advantage of easy manipulation by vegetative propagation methods, resulting in a diverse array of morphological variations [16]. Since chrysanthemum is an important cut flower, breeding is mainly focused on aesthetic characteristics, including floral color and shape. Some other traits, such as long vase life and biotic and abiotic resistance, are equally important for producing a healthy and visually appealing plant. However, breeding efforts to accomplish these traits are comparatively scarce in chrysanthemum because these traits are primarily considered only in the later stages of the breeding line selection process of cultivar development [17]. Conventional breeding mainly utilizes phenotypes to select superior lines, which are often ineffective and laborious, especially for traits such as stress tolerance [18]. In addition to this constraint, some of the most desirable traits, such as blue-colored flowers, which are absent in chrysanthemum, cannot be obtained by breeding strategies owing to technical limitations. Alternatively, molecular breeding, or molecular marker-assisted selection (MAS), provides substantial potential to improve the efficiency of breeding by allowing indirect selection of target traits irrespective of plant growth stage or environmental factors [19]. However, molecular breeding and genetic mapping in polyploid chrysanthemum are advancing at a slower pace owing to the large and complex genome, the requirement of a large number of genetic resources, and scientific limitations, including the requirement of specialized statistical methods to estimate recombination frequency for QTL detection by linkage mapping for development of DNA markers [10,20]. Next-generation sequencing (NGS) and multi-omic technologies provide complete genome sequence information, which accelerates the identification of molecular markers for QTL mapping and GWAS. Transgenic molecular breeding provides a promising platform by transforming plants with foreign genes, and has led to considerable progress in the development of cultivars with desired traits [21]. Recent advances in genome editing technologies have presented significantly greater possibilities for the precise manipulation of genomic sequences of genes of interest, making it a revolutionary tool for application in plant functional genomics and biotechnology [22,23]. Hence, the potential role of these breeding and biotechnology advancements involved in the improvement of various floral attributes of chrysanthemum is discussed in the following sections.

## 3. Plant Architecture

Ornamental plants are grown for attraction and beautifying the environment both outdoors and indoors. Hence, plant architecture is one of the major ornamental attributes and a crucial quality for chrysanthemum breeding and production. Height control is one of the important factors in chrysanthemum, which also influences its harvest. Cultivars with shorter stems generally exhibit higher yields and are economically advantageous for commercial production. Moreover, shorter stems increase quality and commercial value, which is of economic importance for growers [24]. Although culture methods such as thermo-period control and chemical retardants are used to control plant height, they are often inefficient and deleterious to plant growth [24,25]. Overexpression of the tobacco *phytochrome b1* gene in chrysanthemum resulted in shorter plants with larger branch angles [26]. Shorter plant height has been achieved in chrysanthemum by transforming the *Arabidopsis GA-insensitive* (*gai*) gene [27]. Xie et al. (2015) reported that short chrysanthemum varieties could be created by the collective silencing of miniature genes, including *DmCPD* and *DmGA20ox*; the transgenic plants exhibited a dwarf phenotype with delayed flowering [28]. Plant architectural diversity is primarily determined by shoot branching. Branching is an important trait that influences the plant morphology, which determines its aesthetic appeal and economic value, in addition to enabling the plants’ response to environmental stresses [29]. In chrysanthemum, standard-type cut flower production requires manual removal of lateral branches to maintain the plant architecture, which increases the production cost. Disbudding is required to hold a large flower in a single stem; hence, non-branching traits are considered important in standard chrysanthemum production [30]. The regulation of axillary meristem and axillary bud outgrowth reflects the final plant shape; hence, regulation of shoot branching patterns is an important trait for molecular breeding in chrysanthemum. Han et al. (2007) showed that lateral suppressor *Ls*-like antisense gene expression in transgenic chrysanthemum reduced axillary branching [31]. The transgenic chrysanthemum with *DgLsL* showed an enhanced axillary bud growth in sense lines, whereas antisense lines exhibited suppressed axillary bud growth, however there was no significant variation in the number of axillary buds between the control and transgenic lines [32]. In extension to this, Huh et al. (2013), suggested that the manipulation of *Ls* gene alone was insufficient to achieve non-branching chrysanthemum, since the collective regulation of other temperature-dependent genes was required [33]. Transformation of the strigolactone biosynthetic component gene, *DgD27*, cloned from chrysanthemum into *Arabidopsis*, suggested an approach to develop chrysanthemum cultivars with fewer tillers [34]. Dierck et al. (2016) reported that the branching genes, including *CmDRM1*, *CmBRC1*, and *CmMAX1*, were involved in axillary bud outgrowth, suggesting their utility as markers of bud activity [35]. Furthermore, analyses of genes involved in auxin transport, bud dormancy, and strigolactone biosynthesis suggested the involvement of auxin transport in bud inhibition by strigolactones in chrysanthemum [36]. Overexpression of the chrysanthemum cytokinin biosynthetic gene isopentenyl transferase (*CmIPT1*) in *Arabidopsis*, indicated that it was a positive regulator of branching, and plays a crucial role in regulating lateral branching in chrysanthemum [37]. In order to comprehensively understand the genetic complexity of shoot branching, molecular markers were identified, and the inheritance analysis showed a polygenic inheritance of strigolactone pathway genes *BRC1*, *CCD7*, *CCD8*, and *MAX2*. These markers can be efficiently used in the breeding of plant architectural traits [38]. Peng et al. (2015) mapped 16 loci controlling different branching traits, but many of these were unstable in various environments [39]. Approximately 20 QTLs for seven branching traits have been identified in chrysanthemum with contrasting planting densities, to understand the genetics of density-dependent branching [40]. Recently, Sun et al. (2022) performed GWAS on nine different plant architectural traits and identified four candidate genes, including *PhyB*, *BRH1*, *CPC*, and *bZIP16*, which may be involved in the regulation of plant architecture in chrysanthemum [41].

## 4. Floral Color

**Figure 1:**
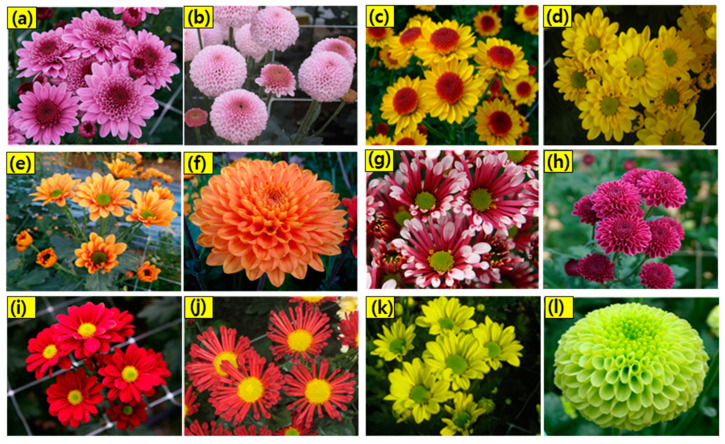
Representative images showing chrysanthemum cultivars with vibrant colors and diverse floral shapes developed by cross breeding at the National Institute of Horticultural and Herbal Sciences (NIHHS), RDA, Korea. (**a**) Glory Pink, (**b**) Pink Bubble, (**c**) Ilweol, (**d**) Yellow Marble, (**e**) Light Up, (**f**) Orange Pangpang, (**g**) Purple Cone, (**h**) Purple Pangpang (**i**) Red Marble, (**j**) 10B1-173, (**k**) Field Green, and (**l**) Green Pangpang.

Among the floral attributes, floral color is remarkably significant, both biologically and economically, in chrysanthemum, which influences the customer choice of cut flowers, pot and garden flowers [42,43]. In addition to imparting aesthetic appeal to the plant, floral color also plays a crucial role in attracting pollinators for reproduction, offering protection against photo-oxidative damage, and providing resistance to biotic and abiotic stresses [44]. The visual quality of chrysanthemum is evaluated based on flower color; hence, color is the major determinant of commercial value [45,46]. Central disc florets of chrysanthemum are usually green or yellow in color, and marginal ray florets exhibit diverse colors of red, orange, purple, green, pink, and white [47]. Although the typical floral color is limited to yellow, white, and pink in wild chrysanthemum, breeding advances have accelerated the development of a wide spectrum of floral colors and patterns in chrysanthemum [9]. Carotenoids, flavonoids, betalains, and anthocyanins are the major pigments responsible for the colorful pigmentation in floral petal coloration [48]. Carotenoids impart orange, yellow, and bright red colors, and anthocyanins include six anthocyanidin types: cyanidin, delphinidin, petunidin, peonidin, malvidin, and pelargonidin. Among these, cyanidin, pelargonidin, and delphinidin are the major types that impart diverse colors ranging from orange to red, and purple to blue [49]. Efforts to develop cultivars with novel innovations in floral color are incessant to meet market demands. Since chrysanthemum is highly heterozygous, conventional crossbreeding between parents with contrasting traits remains effective for breeding new cultivars [3]. Several cultivars have been developed by crossbreeding in chrysanthemum. ‘Lavender Daisy’ with dark purple flower and ‘Dark Bronze Daisy’ with dark bronze ray florets are the novel floral colors in the ‘Mammoth’ series which have been developed by the interspecific cross between *C. weyrichii* (Maxim) × *C. grandiflorum* Tzvelv [50,51]. Various other cultivars, including the standard and spray types, have been bred for novel floral colors. Flower images of various chrysanthemum cultivars developed for floral color from our work are shown in Figure 1 [52,53,54,55].

Recent progress in sequencing technologies has resulted in the whole-genome sequencing of wild diploid chrysanthemum species, *Chrysanthemum nankingense*, and *Chrysanthemum seticuspe* [56,57]. This has accelerated studies on linking genotype with phenotype, thus, overcoming the limitations of molecular breeding in chrysanthemum. Recently, chromosome-level genome sequences have been used as a reference for chrysanthemum breeding and genome editing [58]. The first integrated ultra-dense linkage map in a hexaploid species including chrysanthemum with polysomic inheritance was developed, which has enabled the identification of QTLs for major floral traits, including floral color, flowering time, disc-floret degreening, and ray floret number. Two regions, CLG5 and 7, were significantly associated, and one region, CLG9, may also be associated with floral color. Its associated alleles might be required for the development of pink-colored flowers [59]. Although initial genome-wide association studies (GWAS) for chrysanthemum identified four floral traits, including floral color, floral shape, cultivated type, and ray floret type, these SNPs were not associated with floral color [60]. Recent studies have reported the first simple GWAS-based marker development system for chrysanthemum to identify DNA markers of carotenoid pigmentation in ray petals. The resulting SNP markers developed for each allele were strongly associated with carotenoid cleavage based on the presence or absence of carotenoid-related genes *CmCCD4a-1* and *CmCCD4a-5*, indicating the success of this GWAS [20]. This study presented a breakthrough in chrysanthemum breeding to integrate phenotypes and genotypes for desirable ornamental traits.

Transgenic technology offers the possibility of development of innovative and desirable characteristics in ornamental plants using genes from different plant species. Alterations in floral color and petal pigmentation patterns are regulated by changes in the expression of anthocyanin biosynthetic genes. Although transgenic chrysanthemum overexpressing *MYB1* from *Raphanus sativus* showed increased expression of anthocyanin biosynthetic genes *F3H*, *DFR*, and *ANS*, and anthocyanin accumulation, there was no visual increase in floral color in the transgenic plants [61]. The *Chrysanthemum* genus does not synthesize delphinidin-based anthocyanins naturally because of the absence of *F3′5′H*; hence, blue-colored flowers are absent in chrysanthemum. Therefore, developing blue flowers in this plant is difficult by crossbreeding. Hence, *F3′5′ H-encoding* genes in other plants can serve the purpose of delphinidin synthesis [62]. In an attempt to reconstruct the delphinidin biosynthetic pathway, He et al. (2013) overexpressed the *F3′5′H* gene from *Senecio cruentus* and blocked the cyanidin pathway by downregulating the *F3′H* gene, which was unsuccessful, resulting in bright red flowers owing to increased cyanidin synthesis [63]. Violet/blue-colored flowers in chrysanthemum have been obtained by genetic engineering of the delphinidin pathway through the transformation of the chimeric pansy *F3′5′H* gene under floral specific promoters [64]. A similar attempt was conducted by the combined expression of chrysanthemum *F3H* promoter-driven *alcohol dehydrogenase* (*ADH*) translational enhancer-fused *Campanula F3′5′H*, which resulted in the production of blue/violet flowers [65]. A true-blue chrysanthemum, which is a highly desired ornamental trait, was successfully achieved by transforming the butterfly pea *uridine diphosphate* *(UDP)-glucose–anthocyanin 3′,5′-O-glucosyltransferase* gene and the co-expression of Canterbury bells’ *F3′5′H* [66]. Studies on the regulatory mechanisms revealed that *CmMYB5-1*, *CmMYB6*, *CmMYB7-1*, and *CmbHLH24* were involved in the regulation of light-induced anthocyanin accumulation in chrysanthemum [67]. *CmbHLH2*, along with *CmMYB6*, were found to be involved in the upregulation of *CmDFR*, thereby inducing anthocyanin biosynthesis, suggesting the significance of *CmbHLH2* [68]. Transcriptome analysis of ray florets of *C. morifolium* ‘Jinbeidahong’ under artificial short-day and natural short-day conditions revealed that the light signaling regulator, *ELONGATED HYPOCOTYL5*, was significantly upregulated on natural short days, suggesting that light-induced anthocyanin biosynthesis is tightly regulated by the photoperiod [69]. *CmMYB9a* triggered anthocyanin pigmentation in chrysanthemum flowers by positively regulating *CmCHS*, *CmDFR*, and *CmFNS* [70]. Wang et al. (2022) recently reported a novel MYB transcription factor, *CmMYB21*, which regulated color fading in chrysanthemum by repressing anthocyanin biosynthesis [71].

## 5. Floral Scent

Floral scent, in addition to floral color, is a crucial trait in ornamental crops. Fragrant flowers with floral scents are highly desirable for major floricultural crops [72]. Floral scents emit volatile organic compounds (VOCs) that impart a specific odor. Floral volatiles contribute to reproductive success by attracting pollinators and are also involved in adaptive roles including defense mechanisms against abiotic stresses and repellents [73,74]. Floral VOCs are widely used in cosmetics, perfumes, flavors, and therapeutics. Floral scent is determined by a combination of VOCs that are mainly classified into terpenoids, phenylpropanoids, and fatty acid derivatives from various biosynthetic pathways [75]. Terpenoids are the major floral volatiles that are mainly used by plants for pollination and chemical defense as repellents [76,77]. Although floral fragrance is a crucial trait, floral scent biochemistry is relatively poorly understood compared with the visual cues of floral color and phenotype, which is attributed to the complexity of its quantification, requiring specialized equipment [78,79,80]. However, accumulating evidence in the past decade has led to the identification of novel floral volatiles and several genes that regulate scent. Breeding for floral scent has been scarce because of its complicated heredity patterns, which can be lost or acquired across generations [81]. Approximately 200 types of volatile compounds have been identified in the flowers of chrysanthemum cultivars, the main compounds of which include monoterpenes and oxygenated monoterpenes, including camphor, chrysanthenone, myrcene, α-pinene, eucalyptol, camphene, β-phellandrene, and safranal [82]. These VOCs contribute to the floral scent characteristics and antioxidant activity of chrysanthemum flowers [83]. Monoterpenes and sesquiterpenes are the major herbivore-induced terpenes, and their enhanced emission induces plant defense mechanisms against herbivores in chrysanthemum [84,85]. Linalool, a monoterpene alcohol with a sweet fragrance, is a component of floral scent and is involved in plant defenses against several herbivores [86]. Overexpression of the linalool/nerolidol synthase gene, *FaNES1*, in the plastids of chrysanthemum showed an initial attraction of western flower thrips by the sweet smell of emitted linalool in the transgenic chrysanthemum, followed by the deterrence of these co-attracted herbivores, therefore, representing the ‘attractive smell’ and ‘poor taste’ imparted by the enhanced linalool and linalool glycosides [87]. Sasaki et al. (2017) generated an EST database containing a considerable number of TFs and genes involved in terpene biosynthesis, that regulate floral scent [88]. Furthermore, sensorial and gas chromatography analysis of the floral scent of chrysanthemum cut flowers revealed that monoterpenes such as β-pinene, β-cis-ocimene, and linalool are the major VOCs, and their abundance and concentration are not related to the human nasal perception of floral scent [89]. A recent study by Zhang et al. (2021) on chrysanthemum and its wild relatives revealed that phyllaries and receptacles are the main contributors to VOC accumulation, whereas disc florets are the major contributors to VOC emissions, compared with ray florets [90]. Moreover, wild varieties showed higher concentrations of floral terpenoids than the cultivated chrysanthemum cultivars, and that a smaller capitula emitted enhanced concentrations of floral terpenoids. Although chrysanthemum is a popular cut flower, the molecular mechanisms regulating VOCs have not been elucidated. A deeper understanding of the VOC biosynthetic pathways would provide scope for the development of novel aromatic chrysanthemum cultivars.

## 6. Vase Life

After harvest, cut flowers are usually distributed without roots, and hence are very difficult to maintain and store without losing quality. Cut flower quality is determined quantitatively by weight, stalk length, number of leaves, and flower size, in addition to qualitative characteristics such as flower color, fragrance, physical damage, disease, and freshness [91]. Unlike other horticultural crops, flowers are highly perishable, leading to significantly higher postharvest losses during the export of fresh flowers to foreign markets [92]. Major limitations of cut flower quality and their marketing include postharvest senescence and organ loss, and efforts are being conducted to improve cut flower quality by developing postharvest treatment strategies [93]. Long vase life and survival for several weeks are the major characteristics of the postharvest quality of cut flowers, which include resistance to senescence-inducing factors including ethylene and bacterial infection [14]. Usually, post-harvest chemical treatments are used to increase the shelf life of cut flowers [3]. Fanourakis et al. (2022) suggested that cultivar variations in the oxidative state were mostly responsible for the cold-storage-induced decline in vase life of chrysanthemum cut flowers, whereas the water relations of the cut flower are not clearly involved [94]. Although post-harvest handling techniques can minimize losses, they are not permanent solutions. Hence, enhancing vase life is challenging for breeders, and various biotechnological techniques are being used to achieve this goal. Genetic manipulation of ethylene biosynthesis, perception, and signal transduction leads to an extended shelf life, either by blocking ethylene biosynthesis or ethylene perception. Exposure of chrysanthemum to ethylene causes yellowing of leaves, which manifests even before senescence, resulting in diminished quality, attractiveness, and shortened vase life [92]. Previous reports have shown that leaf yellowing in chrysanthemum is induced by ethylene [95]. Transformation and characterization of chrysanthemum cultivar ‘Sei-Marine’ with mutated ethylene receptor gene *mDG-ERS1*, revealed the role and possibility of these genes in reducing ethylene sensitivity in chrysanthemum [96]. Furthermore, transgenic chrysanthemum with mutated *mDG-ERS1* (*etr1-4*), an ethylene receptor gene, suppresses leaf senescence [97]. The molecular mechanisms involved in the regulation of post-harvest senescence induced by ethylene remain unclear. Hence, efforts have been made to understand these molecular mechanisms through advanced transcriptomic analyses. A recent study on the gene expression regulatory networks in the leaves of post-harvest cut chrysanthemum ‘FenDante’ revealed the involvement of various pathway-related genes, including plant hormone biosynthesis, chlorophyll synthesis and degradation, aquaporins, and senescence-related transcription factors, thus, providing valuable information for further understanding the molecular mechanisms underlying ethylene-mediated leaf senescence in cut chrysanthemum [98].

## 7. Flowering Time and Development

Flowering time is a key trait that significantly influences the planting area, total productivity, and commercial value. The transition from the vegetative stage to reproductive growth is the most critical event, and is triggered by both endogenous and environmental cues [99]. The flowering time usually represents the number of days between the plant transplantation date and the initial flowering day. Accurate information on flowering time is crucial for breeding and commercial production of plants [100]. In chrysanthemum, flowering time is divided into three groups: early, medium, and late [101]. Chrysanthemum is a short-day plant, which starts flowering in the fall when the day length is shorter than 12 h, and the summer plants flower on both short and long days [102]. To meet the commercial demand for chrysanthemum, various techniques, such as shading in summer, have been employed to regulate flowering; however, it is laborious and increases production costs [103]. Since flowering time is considered the major determinant to successful commercial plants, advancing flowering time and enabling the plants to produce flowers even during long days significantly reduces production costs; hence, this trait in chrysanthemum is the major target for breeding and genetic engineering. Various new chrysanthemum cultivars with different flowering times have been developed and molecular advances have been employed to regulate flowering. MADS-box genes are crucial players in floral development as they control floral organ development and flowering time. Among them, the *AP1* gene, for example, is involved in flowering [104]. Overexpression of the *AP1* gene in chrysanthemum led to early bud initiation, 14 days prior, compared with non-transgenic plants during long days. Flowers of transgenic plants exhibited an earlier color configuration and complete inflorescence opening compared with control plants [105]. Flowering Locus T-like (FTL) paralog from *Chrysanthemum seticuspe*, *CsFTL3*, functions as a photoperiodic regulator of flowering. Constitutive expression of *CsFTL3* in chrysanthemum resulted in the development of flower buds under non-inductive long-day conditions. Ectopic expression of *CsFTL3* in *Arabidopsis* led to early flowering in transgenic plants compared with wild-type plants under short-day conditions [106]. Similarly, *CmFTL2* was significantly induced, relative to *CmFTL3*, by sucrose treatment, and was actively involved in floral transition, thus, regulating photoperiodic flowering under short-day conditions [107]. However, the function of *CmFTL1* in summer chrysanthemum is unknown, and when it was constitutively expressed in the short-day cultivar ‘Jinba’, it showed weak florigenic activity [108]. The BBX family of proteins forms a crucial link between the circadian clock and the floral integrator, *FT-BBX24*, a zinc finger transcription factor that modulates both flowering time and abiotic stress tolerance in chrysanthemum. Transgenic lines with suppressed *CmBBX24* (Cm-BBX-RNAi) flowered earlier than the wild-type plants and showed reduced tolerance to freezing and drought stress [109]. Age-dependent regulation of SPL TFs by *miR156* influenced flowering by controlling the expression of *CmNF-YB8* in chrysanthemum [110]. Constitutive overexpression of the *CmERF110* TF from chrysanthemum in transgenic *Arabidopsis* accelerated flowering by approximately 7 days, compared with the wild-type plants [111]. Recent studies have led to the identification of genes related to flowering time, including *CmTFL1c (TERMINAL FLOWER 1)*, *CmMET1 (DNA METHYL TRANSFERASE 1)*, and two miniature related genes, *DmCPD* and *DmGA20ox*, in chrysanthemum [28,112,113]. Constitutive overexpression of *CmTFL1a* resulted in the delaying of the transition to the reproductive phase and significantly affected plant morphology in chrysanthemum cv. Jinba [114]. Nakano et al. (2020) demonstrated that photoperiodic variations in heat sensitivity fluctuations affected flowering in chrysanthemum, supporting an earlier study wherein heat-induced flowering delay was caused by the suppression of *FTL3* expression, suggesting that daily fluctuations in heat sensitivity may correlate with the rhythm of *FTL3* regulation [115,116]. Controlling night temperatures during summer exerts a significant influence on the flowering time and quality of chrysanthemum [117]. A 4 h supplementation of night-interrupting blue light during the photoperiod enhanced flower bud formation and promoted flowering, providing an alternative strategy for artificial short-day induction for flowering during long days [118]. Wang et al. (2020) isolated and characterized the *CmBBX8* TF, and its overexpression was found to accelerate flowering, with upregulated photoperiod-associated genes during both short and long days [119]. However, artificial microRNA (amiR)-enabled knockdown of *CmBBX8* resulted in delayed flowering and downregulation of photoperiod genes, suggesting that *BBX8-FT* regulation is critical in the flowering of summer chrysanthemum. *CmBBX29* has been isolated and characterized from the chrysanthemum ‘Jinba’, and its constitutive overexpression in *Arabidopsis* showed a delay in flowering by suppressing flowering genes, indicating that *CmBBX29* regulates flowering time by the integration of *FLC* and the photoperiod pathway [120]. The R2R3 MYB TF *CmMYB2* was identified as a component of the switching mechanism from the vegetative to the reproductive phase. Overexpression of *CmMYB2* in chrysanthemum induced early flowering, while *CmMYB2* knocked-down plants showed delayed flowering relative to wild-type plants. Both overexpressed and knocked-down transgenics showed different levels of transcription of photoperiod, gibberellin synthesis, and signaling genes, compared with the wild type. This study revealed an interaction between *CmMYB2* and *CmBBX24* that regulates flowering by influencing gibberellin synthesis [121].

## 8. Floral Anatomy and Development

Development of novel floral figure in ornamental plants, such as chrysanthemum, is a constant requirement to enhance its market value. Growth and development of floral organs are key characteristics of ornamental plants. Ray petal size and floret number determine the shape of chrysanthemum flowers. The diversity in chrysanthemum flower shapes and sizes makes it an ideal ornamental plant. However, the molecular mechanisms underlying floral anatomy in chrysanthemum remain largely unexplored. Suppression of the *AGAMOUS* gene in *C. morifolium* resulted in the alteration of gynoecium and androecium to corolla-like tissues, which changed the floral shape [122]. Previous studies have reported the involvement of *CYCLOIDEA* (*CYC*)-like genes in the regulation of flower symmetry [123]. Huang et al. (2016) identified six *CmCYC2* genes with localized expression in ray florets of petals in *C. lavandulifolium* [124]. However, overexpression of *CmCYC_2_c* in chrysanthemum resulted in enhanced ray floret length and number of flowers per plant. There was no significant change in the flower shape, suggesting the complexity and polygenic inheritance of this trait in chrysanthemum. Liu et al. (2021) identified two *Cyc2CL* genes, *Cyc2CL-1* and *Cyc2CL-2*, which play important roles in stamen and ray floret development in chrysanthemum [125]. A recent study proposed the molecular regulation of the evolutionary shift from a radiate to a disciform capitulum in chrysanthemum through the dysfunction of *CYC2g* orthologs [126]. The TCP family of TFs is involved in flower development. *CmTCP20*, from the PCF group in Class I of the TCP TF family, has been isolated and is implicated in petal elongation [127]. Liu et al. (2016) identified approximately 1800 DEGs through transcriptome analysis that included regulatory genes for floral meristem and organ development in chrysanthemum florets [128]. Analysis of petal defects in the bud sport of *Chrysanthemum* ‘Anastasia Dark Green’ revealed a network of genes that regulated the morphology of hooked petals. Ectopic expression of the chrysanthemum polarity gene homolog, *CmYAB1*, reduced petal curvature and flat petals, resulting in round, pompon-like inflorescence in transgenic plants [129]. The anemone type of inflorescence in chrysanthemum is one of the most popular types of inflorescences that exhibit prominent tubular florets with attractive colors. A set of additive and epistatic QTLs have been identified that are seemingly involved in the genetic determination of anemone-type floral forms in chrysanthemum. This study provided a base for identifying the candidate genes for this trait [130]. Floret shape and relative number of florets (flower doubleness) are important traits of chrysanthemum. Corolla tube merged degree (CTMD), representing ray floret shape, and relative number of florets (RNRF), are complex traits that are supported by gene and polygene models [131]. Furthermore, a high-density genetic linkage map revealed three major QTLs for CTMD, and four QTLS controlling RNRF, providing scope for future identification of candidate genes for these traits [132]. Two dCAPS markers associated with capitulum diameter and flowering time have been identified, which have the potential for MAS breeding [133].

## 9. Biotic Stress Resistance

A myriad of microorganisms, such as bacteria, viruses, and fungi, infect ornamental plants, severely affecting their growth and phenotype, leading to reduced commercial value. Pathogens that infect ornamental plants, which are of great significance, cause huge economic losses. The visual appearance of disease symptoms and the pathogen’s impact on growth and morphology limit the visual appeal of the ornamental plant, eventually reducing its market value [134]. Inadequate pathogen-resistant genetic resources, genome complexity, and hexaploidy of chrysanthemum hinder the breeding of biotic stress resistance [13]. Development of cultivars with enhanced tolerance is an important goal for breeders. Various fungal diseases, such as leaf spot, powdery mildew, gray mold, and rust, affect chrysanthemum. Overexpression of *polygalacturonase-inhibiting protein (PGIP)* from *Prunus mumei* in transgenic chrysanthemum enhanced tolerance to *Alternaria* leaf spot [135]. *hrp* genes encode pathogen molecules called hairpins, which activate signaling cascades and induce disease resistance. Overexpression of the *hrp* gene, *hpaG_X00_*, imparted resistance to *Alternaria tenuissima* in chrysanthemum [136]. Introduction of rice *chitinase* gene (*chiII*) enhanced resistance to *Septoria obesa*-caused leaf spot in chrysanthemum ‘Snowball’ [137]. Transcriptome analysis in response to black spot disease caused by *Alternaria* sp. revealed important DEGs and overexpression of a candidate gene, *CmNPR1* (*nonexpressor of pathogenesis-related gene 1*), enhanced resistance against black spots in transgenic chrysanthemum [138]. Xin et al. (2021) demonstrated that the *mildew resistance locus O (MLO)* genes *CmMLO17* and *CmKIC* interact, and the suppression of these two genes showed a reduction in the susceptibility to leaf spot caused by *Alternaria alternata*, suggesting that these two genes are involved in the pathways that support fungal growth [139]. Overexpression of the rice *chitinase* gene (*RCC2*) improved resistance to gray mold disease caused by *Botrytis cinerea* in transgenic chrysanthemum [140]. Transgenic chrysanthemum ‘Shinba’ overexpressing N-methyl transferase genes, including *CaXMT1*, *CaMXMT1*, and *CaDXMT1* showed improved tolerance to *B.* *cinerea* through delayed occurrence of disease and reduced disease index [141]. White rust, caused by *Puccinia horiana* Henn. is a devastating fungal disease in chrysanthemum. Overexpression of a modified sarcotoxin *IA* gene from *Sarcophaga peregrina (msar)* and *Cry1Ab* gene from *Bacillus thuringiensis* showed increased resistance to white rust and tolerance to *Helicoverpa armigera* [142]. A recent study by Bi et al. (2021) demonstrated that the WRKY transcription factor *CmWRKY15-1* is involved in white rust resistance through the regulation of the salicylic acid-mediated disease-resistance signaling pathway in chrysanthemum [143]. Transcriptome analysis has revealed several DEGs in response to blackspot and white rust diseases [144,145]. Lepidopteran insects cause substantial damage to chrysanthemum yield. Transgenic chrysanthemum expressing a modified *δ-endotoxin* gene from *Bacillus thuringiensis* showed tolerance to *Helicoverpa armigera* [146]. Metabolome of thrips-resistant and susceptible chrysanthemum plants were compared by NMR-based metabolomics and identified that the phenylpropanoids, including chlorogenic acid and feruloyl quinic acid, contained inhibitory effects against western flower thrips (*Frankliniella occidentalis*); these are the compounds of choice for insect resistance [147]. Aphids are major pests that damage chrysanthemum by consuming nutrients from the phloem sap and are vectors of various viruses. Xia et al. (2015) performed miRNA expression profiling, and identified the involvement of *miR159a*, *miR160a*, and *miR393a* in chrysanthemum aphid infestation [148]. Recently, cloning and overexpression of *CmWRKY53* revealed that this TF contributes to aphid susceptibility in transgenic chrysanthemum [149]. Numerous viruses, including TAV, TSWV, CNFV, and CVB, attack chrysanthemum, leading to huge losses in floral yield. Transgenic chrysanthemum cv Polaris, expressing both sense and antisense viral *nucleocapsid (N)* gene exhibited strong resistance to TSWV with a lack of disease symptoms and absence of viral accumulation, compared with the wild type [150]. Overexpression of the *coat protein (CP)* gene conferred tolerance against CMV [151]. The sense and double-sense CVB *coat protein (CP)* gene in transgenic chrysanthemum improved tolerance to chrysanthemum virus B [152]. Choi et al. (2015) have presented a transcriptome analysis of chrysanthemum associated with three RNA viruses including TSWV, CMV, and PVX [153]. Chrysanthemum stunt viroid (CSVd) and chrysanthemum chlorotic mottle viroid (CChMVd) are two pathogens that cause severe crop damage globally. Transgenic chrysanthemum developed using sense and antisense RNA of CSVd showed a stronger resistance to CSVd than wild-type plants [154]. A comparative analysis of local accumulation, intra-leaf movement, and systemic translocation of CSVd in susceptible and resistant cultivar suggested that the major difference between the two is the relatively slower distribution of CSVd in leaves, leading to its delayed cell-to-cell movement in the upper leaves of the resistant cultivar than in the susceptible cultivar [155]. Transgenic chrysanthemum harboring siRNAs targeting the terminal regions of CSVd showed a suppressed development of disease symptoms, although there was no difference in the CSVd replication and propagation between transgenic and wild-type plants [156].

## 10. Abiotic Stress Tolerance

Abiotic stress factors, including nutrient imbalance, drought, salinity, cold, and heat stress, significantly affect chrysanthemum by reducing flower longevity and aesthetic value. The breeding of novel cultivars with improved stress tolerance has been a major requisite. Various genes have been identified and characterized from different sources under stressful conditions. Stress responses to these genes function in various cellular processes, including metabolic biosynthetic pathways, cell proliferation, and transcriptional regulation. However, an inadequate number of genetic resources and stress-tolerant genes in ornamental plants hinder breeding for stress resistance. Several families of TFs, such as *DREB*, *ZIP*, *NAC*, and *WRKY*, play a crucial role in abiotic stress tolerance [157]. Hong et al. (2009) developed transgenic chrysanthemum plants expressing *AtDREB1A* with improved heat tolerance and plant survival at 45 °C for 36 h [158]. DREB A-6 subgroup member *CmDREB6* has been cloned, characterized, and overexpressed in chrysanthemum, which exhibited heat stress resistance by inducing genes involved in heat shock response and ROS homeostasis [159]. *CmCPL1* gene encoding RNAPII CTD phosphatase-like 1, isolated from chrysanthemum showed enhanced tolerance to heat stress when overexpressed. Knockdown of *CmCPL1* showed decreased heat tolerance in chrysanthemum plants [160]. Xing et al. (2021) presented a novel regulatory network regulated by melatonin under heat stress in chrysanthemum [161]. Transcriptome analysis showed that melatonin regulated HSPs, HSFs, crucial genes of secondary metabolite biosynthesis, signal transduction, and hormone metabolism. Overexpression of *CmHSP90.5* cloned from chrysanthemum, resulted in sensitivity and reduction in tolerance of *Arabidopsis* to both heat and salt stress through the negative regulation of ion homeostasis and HSP expression [162]. Low-temperature-tolerant chrysanthemum lines have been developed by overexpressing *AtDREB1A*, and transgenic plants showed normal growth in winter [163]. A temperature-induced lipocalin-1-like gene (*DgTIL1*) was recently identified in chrysanthemum and its overexpression has been shown to improve cold tolerance in transgenic plants. *DgTIL1* interacts with a non-specific lipid transfer protein (DgnsLTP), which enhances ROS and POD accumulation, resulting in cold stress tolerance. The novel post-translational modification of lysine-crotonylation of *DgTIL1* at K72 stabilized DgnsLTP, further enabling cold resistance in chrysanthemum [164]. Similarly, the glutathione peroxidase (*DgGPX1*) gene from chrysanthemum improved cold resistance by increasing GPX activity, thereby reducing ROS accumulation under cold stress in transgenic plants. Furthermore, lysine-decrotonylation of *DgGPX1* at K220 increased GPX activity and improved cold tolerance in chrysanthemum [165]. Overexpression of an MYB family TF from chrysanthemum *DgMYB2* increased cold tolerance by regulating the *DgGPX1* gene and reducing ROS accumulation [166]. Overexpression of the bZIP TFs, *DgbZIP3* and *DgbZIP2* increased cold tolerance in chrysanthemum. *DgbZIP3* interacted with another TF, *DgbZIP2*, which further regulated the expression of *DgPOD*, thereby reducing ROS accumulation and increasing tolerance [167]. Tian et al. (2022) identified and characterized 23 TCP TF genes in *C.* *nankingense*, and the expression profiles of these *CnTCP* genes were downregulated under cold stress [168]. Overexpression of one of the TCP genes, *CnTCP4*, in Arabidopsis, induced hypersensitivity to cold stress. Constitutive overexpression of the *CcSOS1* gene encoding the Na^+^/H^+^ antiporter induced salinity tolerance in chrysanthemum cv. Jinba [169]. Overexpression of *CmHSF4* enhanced salt tolerance by maintaining K^+^ concentration, thereby limiting Na^+^ accumulation in chrysanthemum [170]. Various WRKY family TFs have been reported to be involved in salt stress. Overexpression of *DgWRKY4* and *DGWRKY5* improved salinity resistance in transgenic chrysanthemum seedlings and plants [171,172], whereas *CmWRKY17* negatively regulated salt stress tolerance in transgenic Arabidopsis and chrysanthemum plants [173]. Transgenic chrysanthemum plants overexpressing TF *DgNAC1* showed a higher accumulation of POD, SOD, and CAT, resulting in improved salt tolerance [174]. Overexpression of aquaporin genes from *C. morifolium* induced salinity stress tolerance in chrysanthemum [175]. Wang et al. (2021) identified and characterized the *NAC* family of TFs involved in salt stress tolerance in *C. nankingense* [176]. Transcriptome analysis of the leaves and roots of *C. grandiflora* revealed several candidate genes involved in ion transportation, phenylpropane biosynthesis, and plant hormone signal transduction, which can be used in further studies to improve salt stress tolerance in chrysanthemum [177]. A recent study showed that the heterografted chrysanthemum with *Artemisia annua*, exhibited higher salinity tolerance than self-grafted plants by increasing the accumulation of sugar and proline, downregulating ROS-related genes, and upregulating HSP genes. Therefore, this study provided a basis for large-scale chrysanthemum cultivation in saline soils [178]. Transformation of chrysanthemum with *DREB1A* under two promoters, *35s* and *rd29A*, resulted in enhanced tolerance to water deficiency and salinity stress. Transgenic plants with *DREB1A* under *the rd29A* promoter showed higher tolerance than those with the *35s* promoter [179]. *CgDREB1A* enhanced SOD activity and proline content in chrysanthemum, resulting in improved drought tolerance [180]. Constitutive overexpression of *CdICE1* from *C.* *dichrum* in chrysanthemum induced the regulation of *CgDREB* genes, improved proline content, thereby increasing abiotic stress tolerance, including drought, salinity, and low-temperature stress [181]. Large scale transcriptome analysis of chrysanthemum under dehydration stress identified DEGs that provide a resource of candidate genes regulating the dehydration stress response in chrysanthemum [182]. Li et al. (2018) analyzed the genetic variation in a global collection of dehydration-resistant chrysanthemum cut flower varieties and identified four markers that are strongly associated with drought tolerance [183]. Chrysanthemum *WRKY* genes, *CmWRKY1* and *CmWRKY10*, have been reported to play a crucial role in the drought tolerance of chrysanthemum through regulation of the ABA signaling pathway [184,185]. The *ClCBF1* gene from *C. lavandulifolium* enhanced salt and drought stress tolerance when overexpressed in transgenic chrysanthemum cv. White Snow [186]. Overexpression of the AP2/ERF family TF from chrysanthemum, *CmERF053*, in Arabidopsis resulted in the positive regulation of drought stress tolerance, in addition to inducing lateral root and shoot branching [187]. Overexpression of the TF *DgNAC1* in chrysanthemum alleviated drought stress by improving water relation traits, ROS enzyme activities, and upregulation of stress-responsive genes [188]. Similarly, the NAC TF, *ClNAC9*, from *C.* *lavandulifolium*, positively regulated a variety of abiotic stresses, including drought, salinity, and alkaline stress in transgenic chrysanthemum [189]. The zinc finger family TF, *CmBBX19*, improved drought tolerance when suppressed, and increased drought sensitivity when overexpressed, in chrysanthemum transgenic lines upon suppressed or increased drought sensitivity. *CmBBX19* regulated drought tolerance by interacting with the ABA signaling component, ABF3, in an ABA-dependent manner [190]. A nuclear factor Y (NF-Y) TF from chrysanthemum *CmNF-YB8* negatively influenced drought tolerance by regulating the immediate downstream regulator genes, *CmSHN3* and *CmCIPK6*, thus, altering the leaf stomatal opening and cuticle wax accumulation respectively, ultimately affecting drought tolerance [191]. Constitutive expression of *phospholipase Dα* (*CmPLDα*) improved the alleviation of drought stress in transgenic chrysanthemum by maintaining membrane integrity and water balance [192]. Zhang et al. (2021) characterized the transcriptome of a drought-resistant, endemic plant, *C. rhombifolium*, by identifying a large number of transcript sequences, and established a novel plant genetic resource for drought stress resistance [193]. Transgenic tobacco plants overexpressing *CgbZIP1* from *C. grandiflora* exhibited higher tolerance to drought and salinity stress through an ABA-dependent pathway [194]. Role of various genes that are involved in the improvement of ornamental attributes in chrysanthemum has been briefly listed in Table 1.

## 11. Advances in Genome Editing for Chrysanthemum

Gene editing in chrysanthemum is still in its infancy owing to its huge genome, hexaploidy, and the complication of simultaneous mutations in polyploids, wherein events such as double strand breaks and inducing repair occur at each site independently, thus, lowering the probability of mutations with an increased number of target sites. Currently, genome editing is being evaluated by mutagenesis of the transgene in chrysanthemum to demonstrate the effectiveness of CRISPR/Cas9 using the yellowish-green fluorescent protein (*CpYGFP*) gene. sgRNAs have been designed to target the *YGFP* gene from the marine copepod *Chiridius poppei* in transgenic chrysanthemum plants. The absence of fluorescence signals indicated the inactivation of the *CpYGFP* gene, suggesting the possibility of inducing multiple mutations in the hexaploid chrysanthemum by CRISPR/Cas9 [22]. This study provided a base and future scope of genome editing in complex hexaploid chrysanthemum for improving beneficial ornamental traits. However, polyploids such as chrysanthemum require a knockout of all loci of several genes to ensure the possibility of functional overlaps. Hence, Shinoyama et al. (2020) identified complementary DNAs (cDNAs) for *CmDMC1* genes, which were associated with meiotic homologous recombination in chrysanthemum [195]. Transcription activator-like effector nucleases (TALENS), an important genome-editing technology, was used to knock out all six identified *CmDMC1* cDNAs that were found in a specific location on the chromosome. Two chrysanthemum cultivars with the TALEN expression vector resulted in the development of lines with disruption of all *CmDMC1* loci, successfully inducing male and female sterility. This study showed the efficacy of genome editing in the prevention of transgene flow [195]. Since whole genome sequence information of *Chrysanthemum seticuspe* is now available, the scope of genome editing studies in chrysanthemum to improve the ornamental attributes of chrysanthemum should be accelerated in the future [57].

## 12. Conclusions and Future Prospects

**Figure 2:**
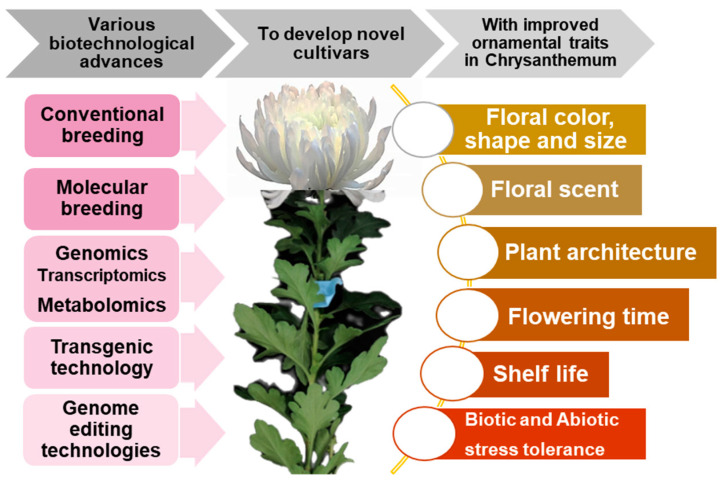
Brief illustration of various strategies and their application in the development of novel chrysanthemum cultivars with improved ornamental attributes.

The ornamental plant industry is developing exponentially, and the increasing demand for ornamental plants has made this industry a profitable sector for plant production. Due to the undisputed influence of the flower market, the annual flower trade increased to EUR 5.6 billion in 2021, from EUR 4.8 billion in 2019 [196]. Since it is a dynamic sector, a constant demand for introducing novel varieties according to consumers’ ever-changing choices, is required. Adapting new technologies to regulate plant growth, development, and production is crucial for the constant expansion of the floriculture industry. Chrysanthemum is one of the top cut flowers globally, with an attractive and diverse floral phenotype. Significant advances have been made in the improvement of ornamental traits in chrysanthemum. However, the utility of advances in molecular technologies remains challenging in chrysanthemum. Molecular breeding of chrysanthemum is lagging owing to its large and complex genome. However, advances in NGS technologies and multi-omic platforms have enabled the availability of complete genome sequences in chrysanthemum, which enable the identification of molecular markers for QTL mapping of desirable traits, a comprehensive understanding of the dynamic molecular regulatory mechanisms, and genetic networks involved in various pathways, thereby enabling the identification of candidate genes useful for genetic engineering. Integration of multi-omic platforms to expedite breeding is still limited in chrysanthemum. Establishing high-throughput phenotyping, developing an integrated platform network for multiple-omics data, and establishing advanced bioinformatic tools and databases to meet data requirements are crucial for the improvement of chrysanthemum breeding. Although breakthrough advances in genome editing technologies such as CRISPR/Cas systems have enormous potential to introduce the desired genetic modifications, its application for chrysanthemum remains in its infancy. However, in addition to the availability of genome sequences, the ease, reproducibility, and high specificity of this breakthrough technology hold immense potential to decipher functional traits, thereby enabling the modification and achievement of target ornamental traits in chrysanthemum. Therefore, this review offers a reference for recent advancements and future exigencies for the promotion of breeding and genetic manipulation of floral attributes in chrysanthemum. A brief illustration of the application of breeding strategies to the development of novel chrysanthemum cultivars with improved ornamental traits is presented in Figure 2.

## Figures and Tables

**Table 1:** Role of various genes involved in the improvement of ornamental traits in chrysanthemum.

Ser. No.	Gene	Source	Resulting Trait	Reference
1	*Phytochrome b1*	*Tobacco*	shorter plant with larger branch angles	[26]
2	*gai*	*Arabidopsis*	shorter plant height	[27]
3	*DmCPD* and *DmGA20ox (silencing)*	Chrysanthemum	dwarf phenotype	[28]
4	*DgLsL (silencing)*	Chrysanthemum	suppressed axillary bud growth	[32]
5	*DgD27*	Chrysanthemum	less number of tillers	[34]
6	*CmDRM1*, *CmBRC1* and *CmMAX1*	Chrysanthemum	axillary bud outgrowth	[35]
7	*CmIPT1*	Chrysanthemum	regulation of lateral branching	[37]
8	*PhyB*, *BRH1*, *CPC* and *bZIP16*	Chrysanthemum	regulation of plant architecture	[41]
9	*F3′5′H*	*Senecio cruentus*	anthocyanin biosynthesis	[63]
10	*F3′5′H*	*Pansy*	anthocyanin biosynthesis	[64]
11	*F3′5′H*	*Campanula*	anthocyanin biosynthesis	[65]
12	*3′,5′-O-glucosyltransferase* and *F3′5′H*	butterfly pea and Canterbury bells	true-blue colored flower	[66]
13	*CmMYB5-1*, *CmMYB6*, *CmMYB7-1* and *CmbHLH24*	Chrysanthemum	light-induced anthocyanin accumulation	[67]
14	*CmbHLH2+CmMYB6*	Chrysanthemum	regulation of anthocyanin accumulation	[68]
15	*CmMYB9a*	Chrysanthemum	regulation of anthocyanin accumulation	[70]
16	*CmMYB21*	Chrysanthemum	repression of anthocyanin biosynthesis	[71]
17	*FaNES1*	Strawberry	floral scent	[109]
18	*mDG-ERS1*	Chrysanthemum	reduced ethylene sensitivity	[96]
19	*mDG-ERS1* (*etr1-4*)	Chrysanthemum	leaf senescence suppression	[97]
20	*AP1*	*Asteraceae*	early flowering	[105]
21	*CsFTL3*	*Chrysanthemum seticuspe*	early flowering	[106]
22	*CmFTL2*	Chrysanthemum	regulation of photoperiodic flowering under short day	[107]
23	*CmBBX24* (silencing)	Chrysanthemum	early flowering	[109]
24	*miR156*	Chrysanthemum	regulation of flowering	[110]
25	*CmERF110*	Chrysanthemum	early flowering	[111]
26	*CmTFL1a*	Chrysanthemum	delayed flowering	[114]
27	*FTL3* (silencing)	Chrysanthemum	heat-induced flowering delay	[116]
28	*CmBBX8*	Chrysanthemum	regulation of early flowering	[119]
29	*CmBBX29*	Chrysanthemum	regulation of delayed flowering	[120]
30	*CmMYB2+CmBBX24*	Chrysanthemum	regulation of early flowering	[121]
31	*AGAMOUS* (silencing)	Chrysanthemum	alteration of floral shape	[122]
31	*CmCYC_2_c*	Chrysanthemum	enhanced ray floret length and number of flowers	[124]
32	*Cyc2CL-1* and *Cyc2CL-2*	Chrysanthemum	stamen and ray floret development	[125]
33	*CmTCP20*	Chrysanthemum	petal elongation	[127]
34	*CmYAB1*	Chrysanthemum	reduction in petal curvature for flat petals	[129]
35	*PGIP*	*Prunus mumei*	tolerance to *Alternaria* leaf spot	[135]
36	*hpaG_X00_*	*Xanthomonas oryzae pv.oryzae*	tolerance to *Alternaria tenuissima*	[136]
37	*chiII*	Rice	tolerance to leaf spot caused by *Septoria obesa*	[137]
38	*CmNPR1*	Chrysanthemum	tolerance to black spot	[138]
39	*CmMLO17*+*CmKIC*	Chrysanthemum	support the fungal growth of *Alternata alternata*	[139]
40	*RCC2*	Rice	tolerance to gray mold caused *Botrytis cinerea*	[140]
41	*CaXMT1*, *CaMXMT1* and *CaDXMT1*	*Coffea arabica*	tolerance to *B.cinerea*	[141]
42	sarcotoxin *IA+Cry1Ab*	*Sarcophaga peregrine* and *Bacillus thuringiensis*	tolerance to white rust and *Helicoverpa armigera*	[142]
43	*CmWRKY15-1*	Chrysanthemum	white rust resistance	[143]
44	*δ-endotoxin*	*Bacillus thuringiensis*	tolerance to *Helicoverpa armigera*	[146]
45	*CmWRKY53*	Chrysanthemum	susceptibility to aphids	[138]
46	*nucleocapsid (N)*	Virus	resistance to TSWV	[150]
47	*coat protein (CP)*	Virus	resitance to CMV	[151]
48	*coat protein (CP)*	CVB virus	resistance to chrysanthemum virus B	[152]
49	*CSVd*	Stunt Viroid	tolerance to CSVd	[154]
50	*AtDREB1A*	*Arabidopsis*	enhanced heat tolerance	[158]
51	*CmDREB6*	Chrysanthemum	enhanced heat tolerance	[159]
52	*CmCPL1*	Chrysanthemum	enhanced heat tolerance	[160]
53	*CmHSP90.5*	Chrysanthemum	sensitivity to heat stress	[162]
54	*AtDREB1A*	*Arabidopsis*	tolerance to low temperature	[179]
55	*DgTIL1*	Chrysanthemum	enhanced tolerance to cold stress	[164]
56	*DgGPX1*	Chrysanthemum	enhanced tolerance to cold stress	[166]
57	*DgMYB2*	Chrysanthemum	improved tolerance to cold stress	[167]
58	*CnTCP4*	*Chrysanthemum nankingense*	hypersensitivity to cold stress	[168]
59	*CcSOS1*	*Chrysanthemum crissum*	tolerance to salt stress	[169]
60	*CmHSF4*	Chrysanthemum	tolerance to salt stress	[183]
61	*DgWRKY4* and *DGWRKY5*	Chrysanthemum	tolerance to salt stress	[172,174]
62	*CmWRKY17*	Chrysanthemum	negative regulation of salt stress	[173]
63	*DgNAC1*	Chrysanthemum	improved tolerance to salt stress	[174]
64	*Aquaporin*	Chrysanthemum	tolerance to salt stress	[175]
65	*DREB1A*	*Arabidopsis*	tolerance to salt and water deficiency stress	[179]
66	*CgDREB1A*	Chrysanthemum	enhanced tolerance to drought	[180]
67	*CdICE1*	Chrysanthemum dichrum	tolerance to drought, salinity and low temperature stress	[181]
68	*CmWRKY1* and *CmWRKY10*	Chrysanthemum	tolerance to drought	[184,185]
69	*ClCBF1*	*Chrysanthemum lavandulifolium*	tolerance to drought and salt stress	[186]
70	*CmERF053*	Chrysanthemum	tolerance to drought stress	[187]
71	*DgNAC1*	Chrysanthemum	tolerance to drought stress	[188]
72	*ClNAC9*	*Chrysanthemum lavandulifolium*	tolerance to drought, salinity, and alkaline stress	[189]
73	*CmBBX19* (silencing)	Chrysanthemum	tolerance to drought stress	[190]
74	*CmNF-YB8*	Chrysanthemum	negative influence of drought stress	[191]
75	*CmPLD* *α*	Chrysanthemum	tolerance to drought stress	[192]
76	*CgbZIP1*	Chrysanthemum	enhanced tolerance to drought and salt stress	[194]

## Data Availability

All the data are contained within the article.
